# Columbia Veterinary College

**Published:** 1883-07

**Authors:** 


Columbia Veterinary College and School of Comparative Medicine.—We are pleased to announce that the above named institution has removed from 217 East 34th Street to its new location, 215, 217 East 37th Street, in this city. The work which has been so efficiently done in the old college buildings during the few years since its organization has been accomplished under very great disadvantages.

The buildings were small and totally unfitted for the work required, and during the last term they were overcrowded.

The present site is centrally located in a quiet street, easy of access from all parts of the city, and in every respect an improvement on the old location.

The property has been purchased at a considerable cost by the President, Dr. Alexander Hadden, for the benefit of the college, and is to be entirely remodelled.

The front building is to be arranged for an office, laboratory, library, small lecture rooms, etc.

The rear building is to be torn down, and a building placed thereon, suitable for a model infirmary, lecture rooms, museum and dissecting room; at an additional cost of about $10,000. When completed according the present plans the college will have a valuable commodious property suitable for the work to which it is dedicated, and so arranged as to be comfortable and attractive to the large and increasing classes which gathered within its walls.

